# Characterizing the blood microbiota of omnivorous and frugivorous bats (Chiroptera: Phyllostomidae) in Casanare, eastern Colombia

**DOI:** 10.7717/peerj.15169

**Published:** 2023-07-06

**Authors:** Nicolas Luna, Marina Muñoz, Adriana Castillo-Castañeda, Carolina Hernandez, Plutarco Urbano, Maryia Shaban, Alberto Paniz-Mondolfi, Juan David Ramírez

**Affiliations:** 1 Centro de Investigaciones en Microbiología y Biotecnología-UR (CIMBIUR), Facultad de Ciencias Naturales, Universidad del Rosario, Bogotá, Colombia; 2 Universidad Internacional del Tropico Americano (Unitropico), Yopal, Colombia; 3 Molecular Microbiology Laboratory, Department of Pathology, Molecular and Cell-Based Medicine, Icahn School of Medicine at Mount Sinai, New York, United States of America; 4 Incubadora Venezolana de la Ciencia, Caracas, Venezuela

**Keywords:** Bats, Microbial communities, Omnivorous, Bacteria

## Abstract

Bats are known reservoirs of seemingly-innocuous pathogenic microorganisms (including viruses, bacteria, fungi, and protozoa), which are associated with triggering disease in other zoonotic groups. The taxonomic diversity of the bats’ microbiome is likely associated with species-specific phenotypic, metabolic, and immunogenic capacities. To date, few studies have described the diversity of bat blood microbial communities. Then, this study used amplicon-based next generation sequencing of the V4 hypervariable region of the 16S-rRNA gene in blood samples from omnivorous (*n* = 16) and frugivorous (*n* = 9) bats from the department of Casanare in eastern Colombia. We found the blood microbiota in bats to be composed of, among others, *Bartonella* and *Mycoplasma* bacterial genera which are associated with various disease phenotypes in other mammals. Furthermore, our results suggest that the bats’ dietary habits might determine the composition and the persistence of some pathogens over others in their bloodstream. This study is among the first to describe the blood microbiota in bats, to reflect on co-infection rates of multiple pathogens in the same individual, and to consider the influence of diet as a factor affecting the animal’s endogenous microbial community.

## Introduction

The ecological features of microbial communities in diverse hosts are determined by intrinsic (age, genetics, sex) and extrinsic (antibiotics, infections and diet) factors. The dietary niche, one of the most important factors regulating host physiology and ecology, modulates structure and ecological relationships of microbial communities (Lozupone et al., 2012). In the case of the gut microbiota, dietary variability is one of the principal mechanisms affecting stability in these communities (Lozupone et al., 2012; Anders et al., 2022). For instance, the type and concentration of nutrients determine the abundance and composition of some bacteria, fungi and protozoa (Yang et al., 2017). Also, the availability of nutrients influences the ecological relationships between symbionts, pathogens and commensals (Lozupone et al., 2012). Thus, the variety of biomolecules from the dietary niche of the host modulates the ecological features and interactions of microorganisms. Although this effect is widely studied in gut microbes, little is known in other communities from other host anatomical zones, such as blood, which harbors a great variety of essential biomolecules from other systems and different cell populations (Castillo et al., 2019).

The circulatory system, considered one of the most sterile systems (Païssé et al., 2016; Castillo et al., 2019), covers different microbial communities that interact with the immune response, red blood cells and nutrients available in the host. These communities, mainly composed of bacteria, are derived from different host physiological systems such as respiratory, intestinal and reproductive (Païssé et al., 2016). Like the intestinal microbiota, the microbial ecology in blood presents dynamic effects associated with the genetic and immune features of the host, the translocation of microorganisms from other organs and the availability of nutrients (Païssé et al., 2016; Castillo et al., 2019). The latter depends on absorption by intestinal cells as they modulate the type and concentration of biomolecules in the bloodstream (Lee et al., 2022), thus regulating the transport and distribution of nutrients. Therefore, the dietary niche could regulate the structure, composition and ecological relationships of different microorganisms in the host’s bloodstream. However, this effect has not been studied profoundly.

Bats are an order of mammals featuring a broad spectrum of ecological tendencies and traits that favor adaptation and colonization to different ecological niches. These traits come from evolution with long-term adaptation to the climates and altitudes they now inhabit (Liu et al., 2015; Jebb et al., 2020; Meng et al., 2021). Also, these ecological adaptations determined that these mammals have fundamental ecological roles, such as pollinators, seed dispersers and indispensable components of the food web for pest control (Kunz et al., 2011; Kasso & Balakrishnan, 2013). Besides their functional role in ecosystems, the evolutionary adaptations in bats allowed them to be reservoirs of potentially-pathogenic microorganisms, mainly viruses (Calisher et al., 2006; Irving et al., 2021), protozoa (Brook & Dobson, 2015; Colunga-Salas et al., 2021), fungi (Zhang et al., 2014; Li et al., 2018) and bacteria (Brook & Dobson, 2015; Allocati et al., 2016). Most of these microbes are identified using specific and unique molecular markers that enable the description of some components of these communities. However, the employment of next-generation sequencing techniques has allowed not only the identification of pathogens in bats but also the composition and structure of the microbial communities present in these mammals (Banskar et al., 2016; Selvin et al., 2019; Li et al., 2020).

As for the microbiota, bats harbor a variety of microorganisms that can range from commensals (Sun et al., 2020; Vanderwolf et al., 2021) to pathogens (Mühldorfer, 2013). All these microorganisms, especially the pathogenic ones, are of interest due to their close relationship with bats and their potential as emerging pathogens (Ingala, Simmons & Perkins, 2018). Regarding microbial ecology, the structure and composition of the microbiota in wild bats varies depending on the biological sample or anatomical sites and the ecological features of the bats (Phillips et al., 2012). The dietary niche of these mammals regulates the intestinal microbial communities and the differential enrichment of metabolic pathways associated with the diet, thereby modulating the composition of symbiotic and zoonotic microorganisms (Banskar, Mourya & Shouche, 2016; Lutz et al., 2019; Ingala et al., 2021). On the other hand, the physiological system or anatomical zone of bats affects the distribution of some microbes, especially viruses (Wille, 2020), regulating their transmission dynamics (Dietrich et al., 2017, 2018).

Although several studies on the ecology of the microbiota in bats have been conducted, where intestinal microbial communities are the most widely analyzed, little is known about the communities of microorganisms present in the blood of these mammals. In this physiological and anatomical system, several studies report the presence of protozoa, such as *Leishmania* (de Rezende et al., 2017), *Plasmodium* (Schaer et al., 2013) and *Trypanosoma* (Jaimes-Dueñez et al., 2020), and zoonotic bacteria, such as *Leptospira* (Silva-Ramos et al., 2022), *Bartonella* (Becker et al., 2018), *Mycoplasma* (di Cataldo et al., 2020) and *Borrelia* (Muñoz-Leal et al., 2021). On the other hand, few studies using next-generation sequencing technologies describe the microbial communities in blood, mainly in parasites, where the co-existence of *Leishmania* and *Trypanosoma* stand out, as well as the role of blood as a source of transmission of zoonotic diseases and the spread of pathogens (Phillips et al., 2012; Patiño et al., 2021). Therefore, it is essential to comprehend the composition of the microbial communities present in these biological samples, given their relationship and impact on public health and pathogen spillover (Letko et al., 2020).

**Figure 1:**
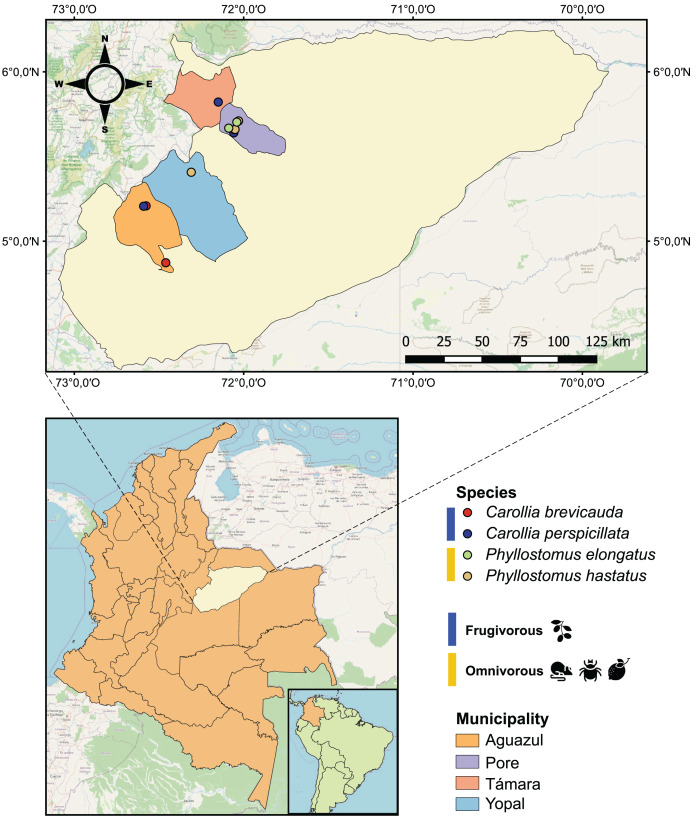
Geographical map of the 25 bat blood samples collected in four municipalities of the department of Casanare, Colombia. Each sample was identified by bat species: *Carollia perspicillata* (*n* = 5), *Carollia brevicauda* (*n* = 4), *Phyllostomus hastatus* (*n* = 14), *Phyllostomus elongatus* (*n* = 2); and classified according to their dietary habits: Frugivorous (*n* = 9) and omnivorous (*n* = 16).

In this study, we describe the prokaryote communities in blood samples of two genera of bats (Chiroptera: Phyllostomidae) with different dietary habits from Casanare in eastern Colombia (Fig. 1) using 16S-rRNA amplicon-based sequencing and the subsequent amplicon sequence variants (ASVs) analysis. We found different potentially zoonotic prokaryotes whose abundance might differ according to the food sources of the bats. These results highlight the co-existence of various potentially zoonotic bacteria circulating in bats blood. Furthermore, these results implicate microbe-microbe and microbe-host interactions in relation with spread of zoonotic diseases.

## Materials and Methods

### Sample collection and processing

During 2022, with the institutional approval of Universidad del Rosario and the Autoridad Nacional de Licencias Ambientales-ANLA-(Resolution N° 01735), 25 bats were captured using mist nets in four municipalities (Pore, Yopal, Aguazul and Támara) in the department of Casanare, eastern Colombia (Fig. 1). All methods were carried out in accordance with relevant guidelines and regulations. The captured bats were anesthetized with ketamine and had 1 mL of blood drawn by cardiac puncture with an insulin syringe. The samples were transferred to guanidine-EDTA buffer for complete blood lysis and DNA preservation. All animals were released once they recovered from anesthesia. Subsequently, these samples were subjected to DNA extraction using the High Pure PCR Template Preparation kit (Roche Life Science, Mannheim, Germany) according to the manufacturer’s instructions.

### Identification of bat species and associated dietary habits

For bat species determination, a 215 bp fragment of the mitochondrial 12S gene was amplified using primers L1085 5′-CCCAAACTGGGATTAGATACCCCC-3′ and H1259 5′-GTTTGCTGAAGATGGCGGCGGTA-3′ in a PCR reaction (Kitano et al., 2007). The amplification profiles included an initial denaturation at 95 °C for 5 min followed by 35 cycles of denaturation at 95 °C for 30 s, annealing at 57 °C for 15 s and extension at 72 °C for 30 s, and finally an extension at 72 °C for 10 min. The PCR products obtained were purified by ExoSAP-IT® and then subjected for Sanger sequencing. The sequences obtained were analyzed with UGENE software and taxonomically assigned by BLAST from the data reported in NCBI (Madden, 2013). Once identified, species were classified according to their dietary habits (frugivorous and omnivorous) (Table 1) using the classification scheme proposed by Ingala et al. (2021). This scheme considers the diets reported in the literature and the food sources of each species in the EltonTraits database (Wilman et al., 2014).

**Table 1:** Analysis of the 10 most abundant genera in the blood microbiota of bats based on their dietary habits.

Comparison	Genus	t	*p*-value	Sig
Frugivorous—Omnivorous	*Mycoplasma*	−9.005	0.001	*
	*Bartonella*	1.732	0.114	
	*Gemella*	−1.665	0.117	
	*Mannheimia*	1.429	0.191	
	*Ottowia*	0.672	0.510	
	*Cloacibacterium*	2.088	0.063	
	*Acinetobacter*	2.335	0.044	*
	*Ottowia*	0.672	0.510	
	*Pseudomonas*	1.840	0.098	
	*Lachnoclostridium*	−0.834	0.415	
	*Streptococcus*	1.074	0.309	

**Note:**

Each genus was analyzed using the Wilcoxon signed-rank non-parametric paired test. Significance codes: “*” 0.05.

### Sequencing and bioinformatics analysis to determine the bacterial communities

We initially verified that the DNA samples complied with the best quality criteria (DNA concentration ≥10 ng/μL and a ratio of A260/280 = 1.8–2.0) for amplicon-based sequencing by an independent entity (Novogene, Bioinformatics Technology Co., Ltd, Beijing, China). The Sequencing was performed according to the following workflow. PCR amplification of the V4 hypervariable region of the 16S-rRNA gene was conducted, which allows genus-level identification of bacteria and archaea, using specific primers (515F and 806R) (Caporaso et al., 2011). The bacterial amplicons were then purified for library preparation using end pairing, the addition of A to tails and ligation of the index adapter. This library was subjected to the sequencing process on a paired-end Illumina platform (Illumina NovaSeq 6000 PE250; Illumina, San Diego, CA, USA) to generate 250 bp paired-end raw reads assuming a minimum expected depth of 100 thousand reads per sample.

After sequencing, raw paired-end de-multiplexed sequences were obtained without primers and adapters. We assessed the quality scores of these sequencing data using FastQC version 0.11.7 (Andrews, 2010). This quality control was consolidated using MultiQC version 1.6 (Ewels et al., 2016). Subsequently, the taxonomic assignment of the amplicon sequence variant (ASVs) was performed using version 1.16 of the DADA2 (Divisive Amplicon Denoising Algorithm) package (Callahan et al., 2016) in R software version 4.0.2 (R Core Team, 2022). For this package, we implemented the recommended parameters of the pipeline for microbiome analysis (https://benjjneb.github.io/dada2/tutorial.html). This pipeline filters individual reads considering a Phred score equal to or higher than 30 to minimize misreads, merges forward and reverse sequences, and infers the amplicon sequence variants (ASVs), defined as the different unique sequences (Callahan et al., 2016), using the central sample inference algorithm. Once the ASVs were obtained, the chimeric structures of the sequences were removed. Finally, with DADA2, each ASV was taxonomically assigned by comparison with the SILVA database version 138.1 (Quast et al., 2012). A minimum confidence bootstrap of 50 was considered for this taxonomic assignment, based on the functions provided by the DADA2 package.

### Blood microbiota composition and diversity metrics

The ASVs corresponding to mitochondrion, chloroplast and eukaryote were filtered from the abundance and taxonomic assignment tables using the R Phyloseq package version 1.40.0 (McMurdie & Holmes, 2013). Then, the ten most abundant phyla and genera of bat blood microbiota were identified considering the dietary habits of bats. This was done based on the proportion of reads of each ASV to the total (relative abundance) of the sample dataset. On the other hand, we generated rarefaction curves to determine the diversity of ASVs of each sample based on the number of reads obtained from the sequencing. These curves were made and visualized with phyloseq (McMurdie & Holmes, 2013), ampvis2 version 2.7.31 (Andersen et al., 2018) and iNext version 3.0.0 (Hsieh, Ma & Anne, 2022) packages. Differences in the abundances of each ASV according to dietary habits were analyzed using Welch’s t-test of the stats package version 4.2.0 (R Core Team, 2022).

To quantify the diversity of ASVs for each dietary habit (alpha (α) diversity), we used the Shannon-Wiener (species diversity) and Simpson (species dominance) indices from microbiome package version 1.18.0 (Lahti & Shetty, 2017) and the rarefaction analysis that conducts iNterpolation and EXTrapolation of iNext package version 3.0.0 (Hsieh, Ma & Anne, 2022). We evaluated the differences obtained between dietary habits using variances and 95% confidence intervals (CI) from diversity estimation based on Hill numbers. In terms of beta (β) diversity, the dissimilarities of the microbiota between dietary habits were assessed and visualized by principal coordinate analysis (PCoA) of the phyloseq package (McMurdie & Holmes, 2013). To assess changes in microbiota communities associated with bat dietary habits, we applied a permutational multivariate analysis of variance test (PERMANOVA), considering the assumptions, from the vegan package version 2.6-2 (Oksanen et al., 2019) with 9,999 permutations. Both PCoA and PERMANOVA analyses were performed on Bray-Curtis distances obtained from the relative abundances of each ASV.

### Analysis of differential microbial genera according to bat feeding habits

To determine the differential microbial genera for each dietary habit, we used the differential analysis of DESeq2 (Love, Huber & Anders, 2014; Cao, 2020). For this analysis, the relative log expression (RLE) was used as a method of normalizing abundance data, the Bonferroni test was used as a method of correcting the significance of the data and a *p*-value cutoff of 0.05. Subsequent to the identification of the differential genera, the sequences of those genera whose species have been associated with different zoonotic diseases were taxonomically assigned at the species level by BLASTn (Madden, 2013). The taxonomic assignment for each genus was performed by comparing the reads with a reference database constructed from 16S-rRNA gene sequences reported in RefSeq (O’Leary et al., 2016), an e-value of less than 10 and a percentage of sequence identity greater than 95%. The information obtained was cross-referenced with the respective abundance values and visualized by means of a sinkplot of the ggalluvial package version 0.12.3 (Brunson, 2020).

## Results

### Identification of bat species and dietary habits

Of the 25 individuals sampled in the localities of the department of Casanare (eastern Colombia), we identified four species of bats: *Carollia perspicillata* (*n* = 5), *Carollia brevicauda* (*n* = 4), *Phyllostomus hastatus* (*n* = 14), *Phyllostomus elongatus* (*n* = 2). Regarding species feeding habits, we found that *P. hastatus* and *P. elongatus* are mainly classified as omnivorous (*n* = 16), and *C. perspicullata* and *C. brevicuada* as frugivorous (*n* = 9) (Fig. 1 and Table S1).

### Quality control of reads associated with ASVs

After sequencing, our samples had between 85,000 and 174,000 raw reads with a Phred quality score above 30 and a sequence size between 216 to 234 bp (Table S2). These data assigned 10,420 ASVs, of which 763 correspond to eukaryotes, chloroplasts and mitochondria. The remaining 9,657 ASVs belonged to 51 phyla and 961 genera of prokaryotes, mainly bacteria (Table S3). On the other hand, the rarefaction curves results demonstrated that the sequencing depth employed was sufficient to analyze the diversity and composition of ASVs present in the bat blood samples (Fig. S1).

### Composition of microbial communities based on dietary habits

**Figure 2:**
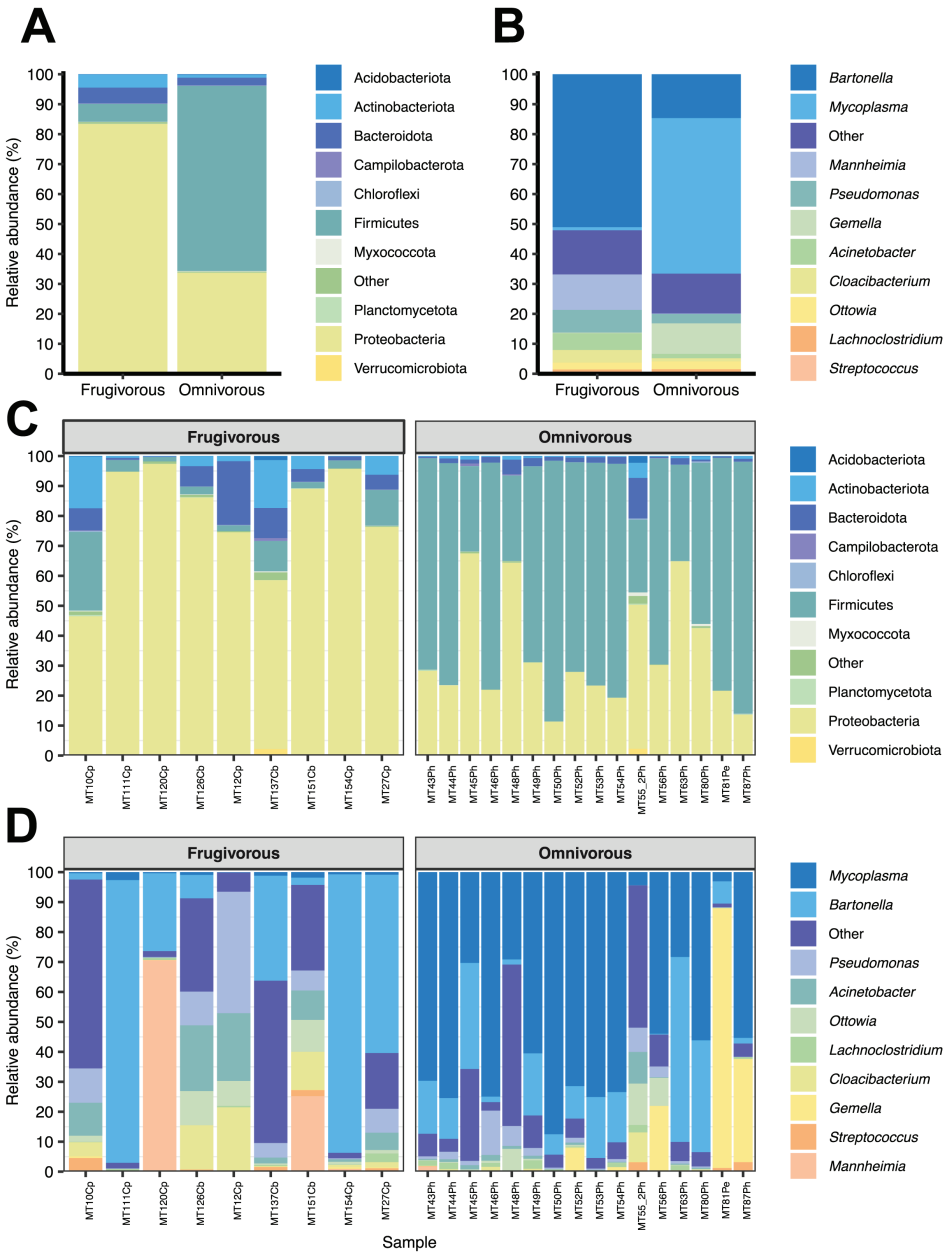
Composition of microbial communities in blood samples of omnivorous and friguivorous bats (Chiroptera: Phyllostomidae). The relative abundance of the ten most frequent (A) phyla and (B) genera of bat blood microbiota. The relative abundance of the 10 most frequent (C) phyla and (D) genera of the blood microbiota of each individual sampled. In each panel, the group “Others” represents the sum of the relative abundances of the other phyla or genera of the blood microbiota of bats.

Of the 51 phyla identified in bat blood microbiota, Proteobacteria and Firmicutes were the dominant phyla (Fig. 2). Concerning their abundance, these phyla vary according to bat dietary habits (Fig. 2A), where Proteobacteria was predominant in frugivorous individuals (~80%) while Firmicutes was prevalent in omnivorous (~60%). At the genus level, *Bartonella*, *Mycoplasma*, *Pseudomonas*, *Mannheimia* and *Gemella* were the predominant groups in the blood microbiota of bats (Fig. 2B). Likewise, the abundances of these genera showed a similar pattern to the phyla, where *Bartonella*, *Mannheimia* and *Pseudomonas* were abundant in frugivorous bats while *Mycoplasma* and *Gemella* were abundant in omnivorous bats (Fig. 2B).

**Figure 3:**
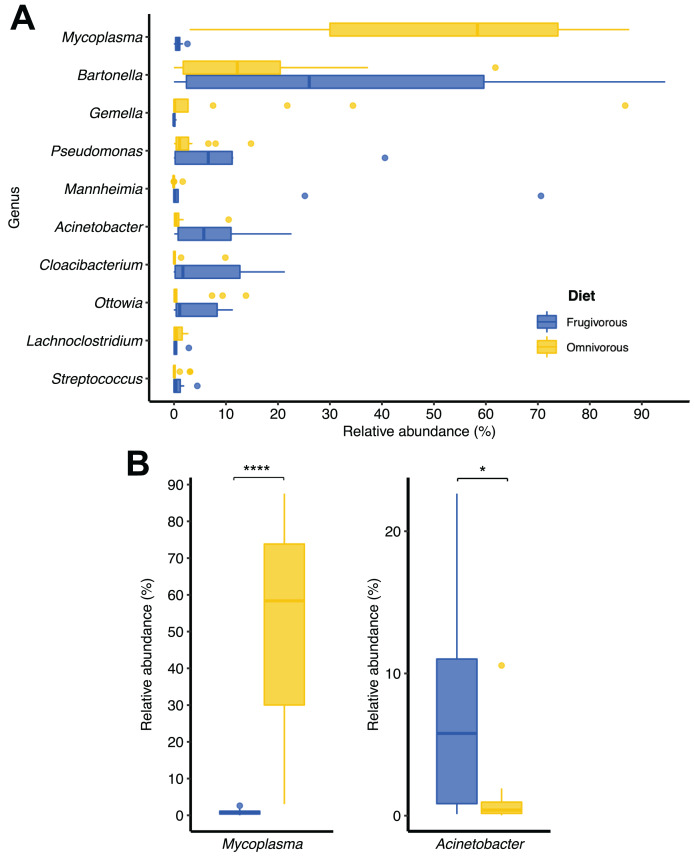
Changes in the relative abundances of the ten most frequent genera according to the dietary habits of bats. (A) Relative abundance of the ten most abundant genera. (B) Relative abundance of significantly different genera by dietary habit. Significance codes: *p < 0.05; **p < 0.01; ***p < 0.001; ****p < 0.0001.

In terms of dietary habits, we found that only the relative abundances of the dominant phyla differ significantly (Firmicutes: *p* = 0.000001958 and Proteobacteria: *p* = 0.00004405; Fig. S2). Likewise, at a finer taxonomic scale, the relative abundances of the 10 most abundant genera varied by this ecological trait (Fig. 3A). This variation was significant only in *Mycoplasma* (*p* = 0.001; Table 1) and *Acinetobacter* (*p* = 0.044; Table 1). In terms of relative abundance, *Acinetobacter* showed higher relative abundance in frugivorous than omnivorous (Fig. 3B). In contrast, omnivores showed a higher abundance of *Mycoplasma* than frugivores (Fig. 3B and Table 1). Even though at the dietary habit level, we observed a dominance of Proteobacteria (*Bartonella*, *Mannheimia* and *Acinetobacter*) and Firmicutes (*Mycoplasma*). At the individual level, we identified variations in relative abundances of these taxonomic groups (Fig. 2C), mainly in the ten most abundant genera (Fig. 2D), where the microbiota of some frugivorous individuals was dominated by *Bartonella* and others by *Mannhemia*. Similarly, in omnivorous bats, the blood microbiota of some individuals was predominated by *Mycoplasma* and others by *Gemella*. Nevertheless, these variations do not modify the previous patterns of abundance observed.

### Alpha and beta diversity metrics

**Figure 4:**
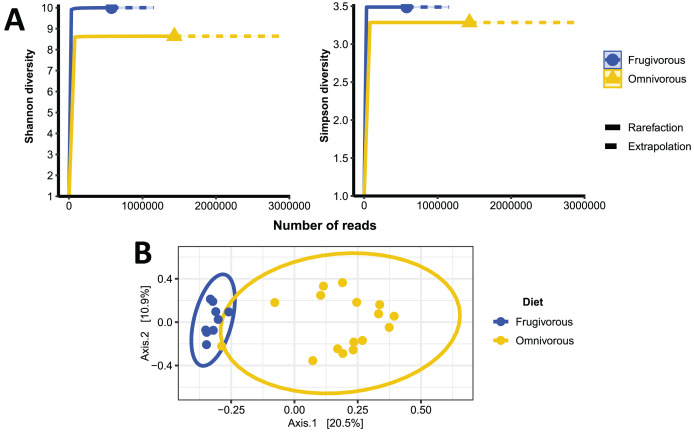
Alpha and beta diversity metrics of bat blood microbiota. (A) Individual-based rarefaction (solid lines) and extrapolation (dashed lines) of microbial’s Shannon and Simpson diversity according to the dietary habits of the bats. Each curve shows the diversity of prokaryote genera (ASVs) in terms of the number of reads with a 95% CI. (B) Principal coordinate analysis (PCoA) based on the dissimilarity of blood microbial communities of omnivorous and frugivorous bats.

The alpha diversity indices (Shannon–Wiener and Simpson) indicate that the blood microbiota in bats does not present dominance of taxonomic groups in their microbial communities (Fig. S3). Furthermore, we found significant variations in these indices based on the dietary habits of these mammals (Table 2 and Fig. 4A). In terms of beta diversity, PCoA analysis shows two clusters associated with dietary habits that differ substantially (Fig. 4B). Besides observing differences in dispersion between clusters, these are not randomly distributed based on their dietary habits (PERMANOVA test: F = 4.6372, *p* = 0.0001, R^2^ = 0.16779, Df of groups = 1, Df of residuals = 23).

**Table 2:** Analysis of the alpha diversity of the blood microbiota of bats based on their dietary habits.

Dietary habit	Alpha diversity measure	Estimator	[CI 95%]
Frugivorous	*Shannon*	10.00	[9.949–10.06]
	*Simpson*	3.488	[3.473–3.503]
Omnivorous	*Shannon*	8.644	[8.612–8.674]
	*Simpson*	3.285	[3.277–3.292]

### Differential microbial genera by bat dietary habits

**Figure 5:**
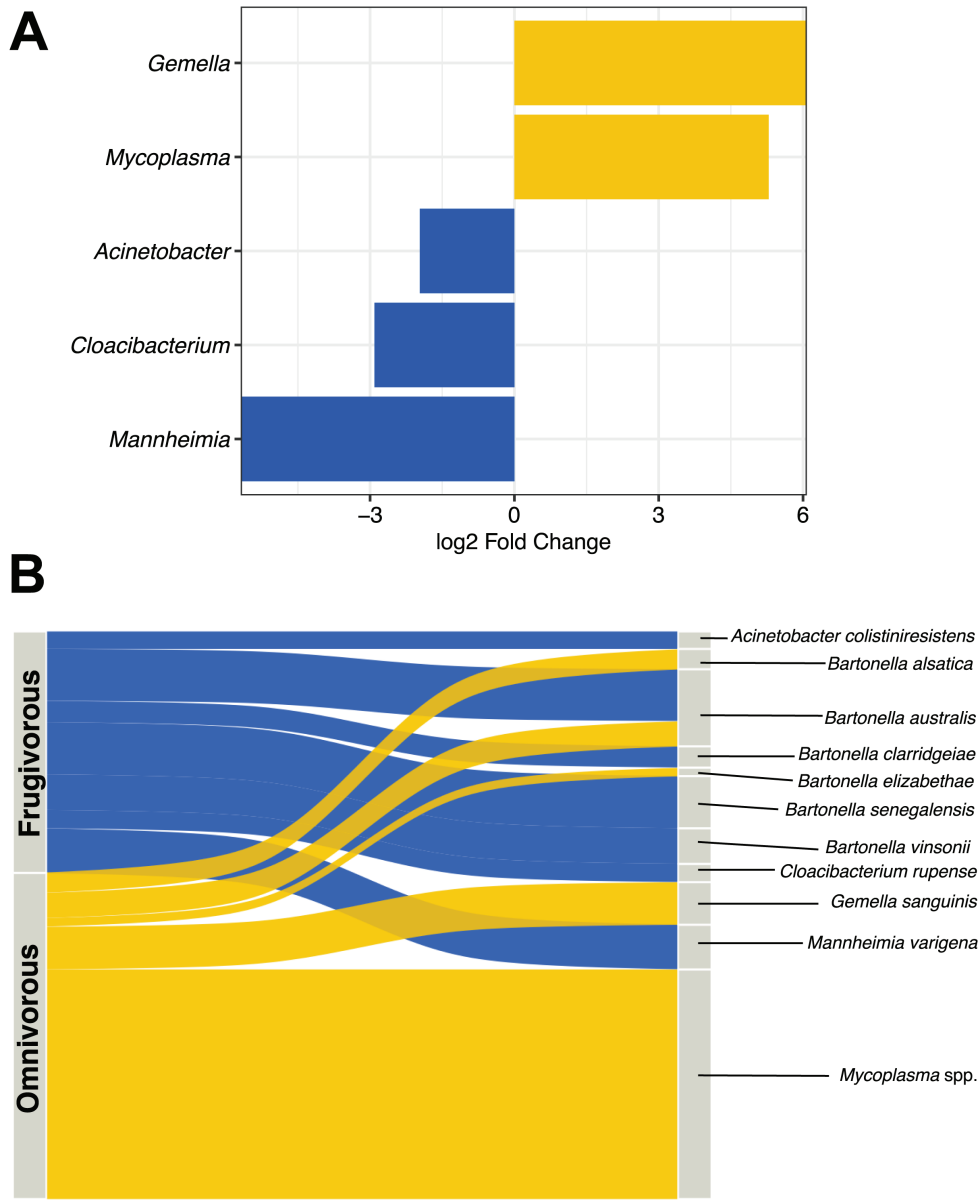
Analysis of differential microbes and pathogenic agents according to bat dietary habits. (A) Differentially abundant genera in the blood microbiota of omnivorous (yellow) and frugivorous (blue) bats according to DESeq analysis. (B) Differential and pathogenic species with a relative abundance greater than 1% in the blood microbiota of bats by their dietary habit. The thickness of the sinkplot indicates the relative abundance corresponding to the genera or species.

DESeq analysis indicated that *Gemella*, *Mycoplasma*, *Acinetobacter*, *Cloacibacterium* and *Mannheimia* were differentially present according to the dietary habit of bats (Fig. 5A). *Gemella* and *Mycoplasma* were genus-specific in the microbiota of omnivorous individuals while *Acinetobacter*, *Cloacibacterium* and *Mannheimia* were differential in frugivorous individuals. Taxonomic assignment of the reads of these differential genera and pathogens (*e.g*., *Bartonella*), showed different species, whose abundance differs based on dietary habits (Table S4). Among the species, the abundances of *Bartonella alsatica*, *Bartonella elizabethae*, *Gemella sanguinis* and *Mycoplasma* spp. were higher than 1% in the blood microbiota of omnivorous bats (Fig. 5B and Table S4). In contrast, in frugivorous bats *Mannhemia varigena*, *Bartonella senegalensis*, *Bartonella clarridgeiae* and *Acinetobacter coliniresistens* predominate with an abundance greater than 1% in the blood microbiota of these bats (Fig. 4B and Table S4).

## Discussion

Diverse microorganisms such as bacteria, have been hypothesized as potential symbionts that regulate the physiology of bats (Lutz et al., 2019; Ingala et al., 2021). In contrast, some taxa, mainly parasites and viruses are known as potential zoonotic microorganisms (Brook & Dobson, 2015). Although these communities are differentially distributed in various anatomical sites of bats (Dietrich et al., 2017; Lutz et al., 2019), raising relative questions about microbe-host interactions, little is known about the ecological structure of microorganisms in other anatomical sites, such as the blood. As for this anatomical zone, few studies have characterized and analyzed microbial communities by amplicon-based sequencing, (Dario et al., 2017; Patiño et al., 2021), especially on prokaryote communities. Herein, we described the prokaryote communities in blood samples from omnivorous and frugivorous bats and analyzed the effect of the dietary niche on the composition of microbial communities.

The composition and diversity in blood microbiota of omnivorous and frugivorous bats indicate variability of bacterial taxa. This result is consistent with the microbial ecology reported in other body fluids (feces, saliva and urine), having a high variability of microorganisms (Carrillo-Araujo et al., 2015; Dietrich et al., 2017; Ingala et al., 2021). Moreover, the diversity and composition patterns herein portrayed differ from those reported in other mammals (Song et al., 2020). This difference, due to the difference in life-history (evolutionary adaptations and ecological traits), such as flight, migration, or dietary niche, allowing bats to host different microorganisms (Song et al., 2020). Even though the relationships between microbial communities and bats may differ from variables such as geography and host phylogeny (Phillips et al., 2012). Most of the variation is mainly explained by ecological and behavioral characteristics such as dietary traits (Phillips et al., 2012; Ingala, Simmons & Perkins, 2018). Moreover, the ecological features of these species, we observed no variation in the clusters when analyzing the geographical location and bat species variables (Figs. S4 and S5) and migration (Karesh et al., 2012) and the distance between the sampled areas (<100 km) could explain the clusters found. However, we were unable to collect blood samples of different dietary habits in many of the municipalities. Therefore, future studies that control sampling should evaluate the ecological and geographical factors shaping the blood microbiota of these mammals.

We observed changes in the relative abundance based on the dietary traits of the bats of different taxonomic groups. These changes in abundance might be determined by the quality and availability of nutrients in blood, related to dietary habits. Previous studies report dietary features as one of the main factors in determining the microbiota of bats (Phillips et al., 2012; Lutz et al., 2019; Ingala et al., 2021); where the diet habits and physiological features modulate the structures of microbial communities through differential expression of metabolic pathways associated with diverse microbial genera (Phillips et al., 2012; Lutz et al., 2019). Therefore supporting our findings, where a possible effect of bat diet might regulate the abundance of microbial communities in blood. For instance, the abundant genera in frugivorous bats are characterized by a high metabolism of carbohydrate-derived sugars (Barbe, 2004). By contrast, the dominant bacteria of omnivorous bats display metabolic pathways associated with protein metabolism (Schuster et al., 2002; García López & Martín-Galiano, 2020). However, further studies are needed to assess this host effect and to determine whether other factors promote this variation.

In terms of the ecological features of the communities of bat blood microbiota, the most abundant bacteria genera (*Mycoplasma*, *Bartonella*, *Acinetobacter* and *Mannhemia*) have been detected in blood as well as other histological samples (spleen and heart) (Correia Dos Santos et al., 2020; Corduneanu et al., 2021; Descloux et al., 2021). In bats, the information is scarce in terms of the functional roles and ecological features of these bacteria, but some species are associated with different zoonotic diseases such as Bartonellosis and Pneumonia (Mühldorfer, 2013; Morris et al., 2019; Komatsu et al., 2019). The several studies that described these zoonotic species used specific tools (PCR or Sanger sequencing) to determine the presence or absence of these microbes (Mühldorfer, 2013; Morris et al., 2019; Komatsu et al., 2019). However, these techniques cannot characterize and/or describe the ecology of these bacteria. Therefore, our results from 16S-rRNA sequencing highlighted not only the ecological features or potential bacteria-bat interactions but also the coexistence of zoonotic genera, an interesting finding especially given that in bats only the coexistence of parasites is documented (Patiño et al., 2021).

We observed a whole diversity of *Bartonella* species in bat blood microbiota. These species have been documented in other bat species (Corduneanu et al., 2018), other mammals such as rodents, cattle, and wild animals (Jiyipong et al., 2014) and ectoparasitic vectors, such as mosquitoes, ticks and fleas (Nabeshima et al., 2022). Moreover, the epidemiology and genomics of these species relate to bats as wild reservoirs associated with the transmission cycle of different *Bartonella* species and genotypes (Sándor et al., 2018; André et al., 2019), independently of the ecological characteristics of the mammals. Thus, abundances found of these hematic bacteria in both omnivorous and frugivorous bats could hint a unique microorganism-bat interaction that deserved further study. In contrast to *Bartonella*, we found that *Mycoplasma* species are most abundant and differential in the blood microbiota of omnivorous bats. Some species of this genus have been found circulating in other bats species (Correia Dos Santos et al., 2020), wild mammals (André et al., 2020) and some ectoparasites such as ticks and mosquitoes (Shi et al., 2019). The biology of mycoplasmas shows close evolutionary relationships dependent on the phylogeny of bats (Becker et al., 2020). Nonetheless, our study did not find a phylogenetic effect of omnivorous bats on *Mycoplasma*; hence the dominance of this genus seems to be more associated with the ecological features of bats than with their phylogenetic relationships. As for *Acinetobacter* and *Mannhemia*, these genera are associated with intestinal and urinary diseases, mainly in livestock (Morris et al., 2019; Komatsu et al., 2019). In bats, interactions and dynamics with these genera are so far unknown. However, the abundance of these circulating zoonotic bacteria in bats is striking given that these are associated with disease outbreaks in livestock (Morris et al., 2019; Komatsu et al., 2019). Therefore, it is possible that bats might be an intermediate host that allows the transmission of these genera. Whether bats play a fundamental role in the epidemiology of these bacteria needs to be further investigated.

Although blood is considered one of the most sterile physiological systems, the presence of microorganisms in bat blood highlights the microbe-host interaction, especially the role of the immune system of these reservoirs. To date, there are two hypotheses under discussion that relate to the microbe-host interaction in this physiological system: (1) An immune dampening, in which the bat has evolved suppressive/inactive inflammatory pathways to blood pathogens that would sustain shock in mammalian circulations (Randolph & Barreiro, 2018). (2) A resistance, describing particularly potent immune responses which allows for mild seroprevalence and states of premonition as a protective mechanism from future infection without exacerbated response (Randolph & Barreiro, 2018). Each of these hypotheses have been supported by analyzing the genetic and cellular components of the immune system in different bat species (Ahn et al., 2016). Nevertheless, these theories leave unanswered questions: is there a similarly hematophagous vector transmitting the pathogen directly to the animal’s blood stream? or is the origin of bacterial transmission from ecological niche to circulation a function of poor surveillance/leaky junctions at the level of gastrointestinal endothelium? From the microbe-to-host perspective now, it may be speculated that successfully persisting pathogens somehow mitigate their own virulence factors allowing them to live within a host that serves as a casual ecological reservoir. This implies that there are still several questions about the microbial ecology of neotropical bats that remain to be answered.

Based on the ecological characteristics and evolutionary adaptations of the bats studied (Karesh et al., 2012). The abundance and circulation of different bacteria, mainly zoonotic, could imply a potential dispersal/transmission of these bacteria in sylvatic and urban environments since the bats studied were captured in the proximity of human settlements (Table S1). On the other hand, according to reports from Global Forest Watch (https://www.globalforestwatch.org/dashboards/country/COL/9/?category=forest-change), in recent years the ecosystems of the localities where the bats were captured presented losses in tree cover as consequence of anthropogenic factors. The habitat loss would imply a displacement of bats to urban settlements. Thus, it leads to a higher probability of the occurrence of spillover processes from bats to other hosts (humans or domestic animals/livestock), whose health may be affected by the transmission of these bacteria. Therefore, we highlight the need for studies focusing on the frequency of associated diseases, vector dispersal and transmission efficiency to provide information on the mechanisms of transmission and spread of these microorganisms and avoid the possible emergence of new disease outbreaks caused by the spread of bats to humans, other mammals or livestock.

The main limitation of the study is the sample size and the distribution of the sampled species, which despite finding patterns associated with ecological features, does not show the effect of environmental or geographical variables. Another limitation is not knowing whether environmental variables and ecological interactions might be contributing to the differentiation of the blood microbiota in bats. Therefore, future studies should include more samples to analyze the different variables that could modulate the blood microbiota of bats. These studies should also describe the viral and eukaryotic (parasite and fungi) communities present in the blood of bats. Finally, a study evaluating the microbiota ecological dynamics in different anatomical sites and fluids is needed. Despite its limitations, this study is the first to describe the bacterial communities in bat blood and the possible role of dietary habits on the structure and diversity of these microbes.

## Conclusions

In summary, the blood microbiota of omnivorous and frugivorous bats is composed of different potentially pathogenic bacterial genera, such as *Bartonella* and *Mycoplasma*, that might depend on the ecological and physiological features of the host. Furthermore, the abundances of these communities might differ according to the bat’s food sources, which could influence the prevalence of the microbiota genera. Further, metagenomic and metabolomics studies coupled with epidemiological data are required, which will provide information on the microbial species, associated metabolites, virulence factors and the ecology of the genera present in the blood microbiota. This study presents as limitations the low sample size, geographical restricted and the lack of complementary estimations of oral and fecal microbiota from the same individual. In turn, future studies should circumvent these limitations including other dietary habits (insectivore, carnivore and hematophagous) and extend the description of microbial communities to parasites, viruses, and fungi.

## Supplemental Information

10.7717/peerj.15169/supp-1 Supplemental Information 1 Rarefaction curves of the bats analyzed by (A) samples and (B) dietary habit. Each curve shows the diversity of prokaryote genera (ASVs) in terms of the number of reads in each sample.

10.7717/peerj.15169/supp-2 Supplemental Information 2 Changes in the relative abundances of the phyla Proteobacteria and Firmicutes according to the dietary habits of bats.

10.7717/peerj.15169/supp-3 Supplemental Information 3 Beta diversity metric of bat blood microbiota considering the geographic locality of the sample.

10.7717/peerj.15169/supp-4 Supplemental Information 4 Beta diversity metric of bat blood microbiota considering bat species.

10.7717/peerj.15169/supp-5 Supplemental Information 5 Beta diversity metric of bat blood microbiota considering bat species.

10.7717/peerj.15169/supp-6 Supplemental Information 6 Information associated with each of the blood samples collected in different localities of the department of Casanare.

10.7717/peerj.15169/supp-7 Supplemental Information 7 Count and length of reads obtained by amplicon-based sequencing.

10.7717/peerj.15169/supp-8 Supplemental Information 8 Count of the number of reads, ASVs, phyla and genus found in archaea and bacteria.

10.7717/peerj.15169/supp-9 Supplemental Information 9 Relative abundances of the species of the differential genera from dietary habits of bats. Each species was identified by BLAST with an identity percentage of 97%.

## Additional Information and Declarations

### Data Availability

The following information was supplied regarding data availability: The datasets generated and/or analyzed during the current study are available at ENA: PRJEB53500.
