# Supplementary figures and images for: Characterizing the blood microbiota of omnivorous and frugivorous bats (Chiroptera: Phyllostomidae) in Casanare, eastern Colombia
Source: PeerJ. 2023 Jul 6;11:e15169. doi: 10.7717/peerj.15169 (PMC10329821; doi:10.7717/peerj.15169)

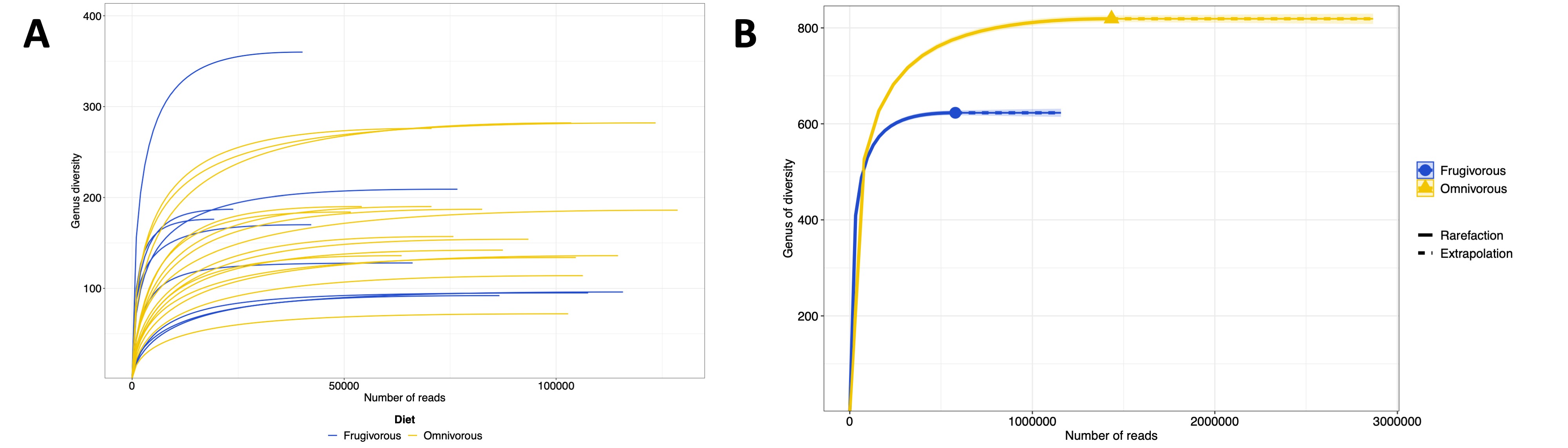

Supplement: Supplemental Information 1 — Each curve shows the diversity of prokaryote genera (ASVs) in terms of the number of reads in each sample. [file peerj-11-15169-s001.jpg]

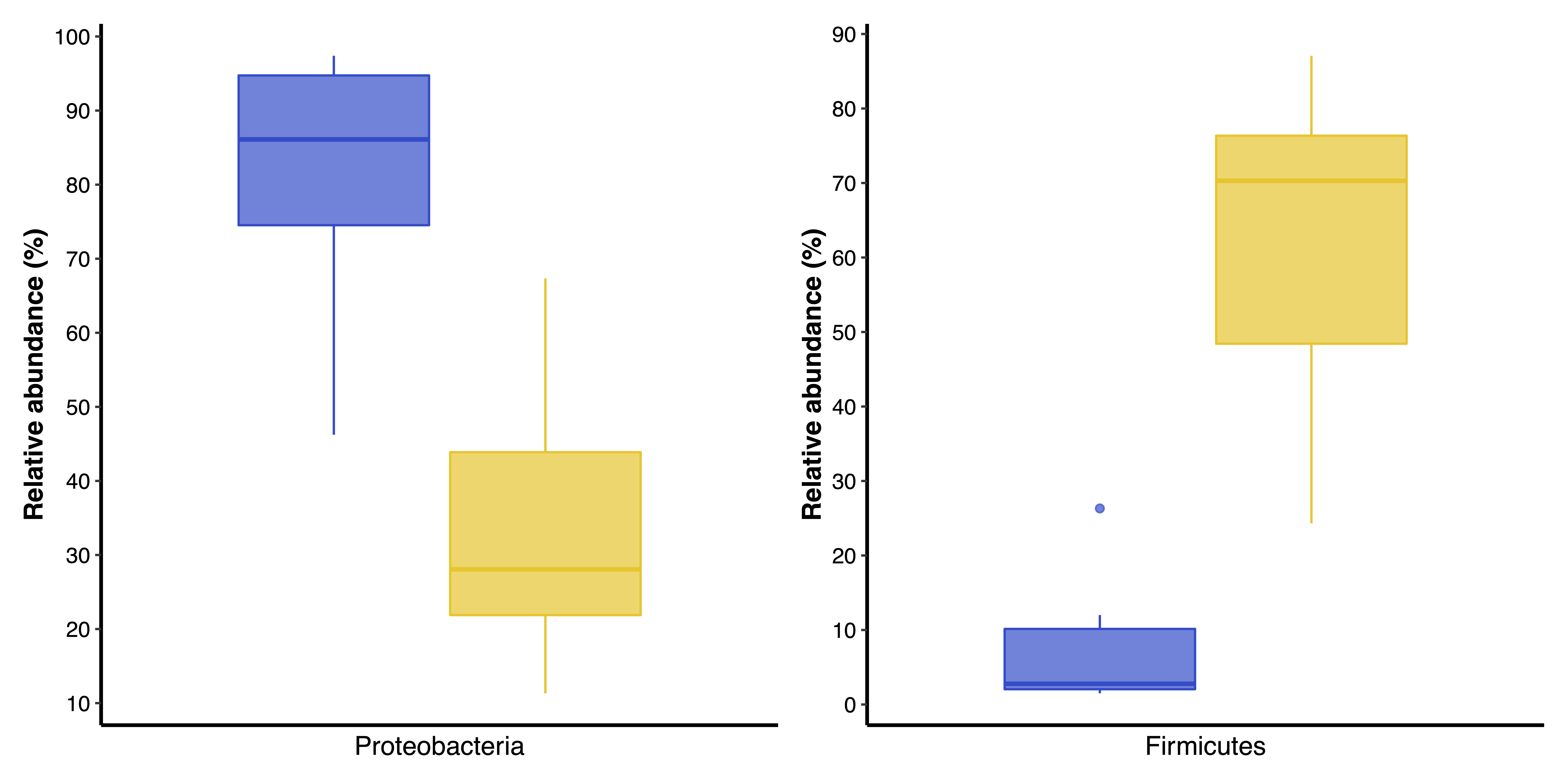

Supplement: Supplemental Information 2 [file peerj-11-15169-s002.jpg]

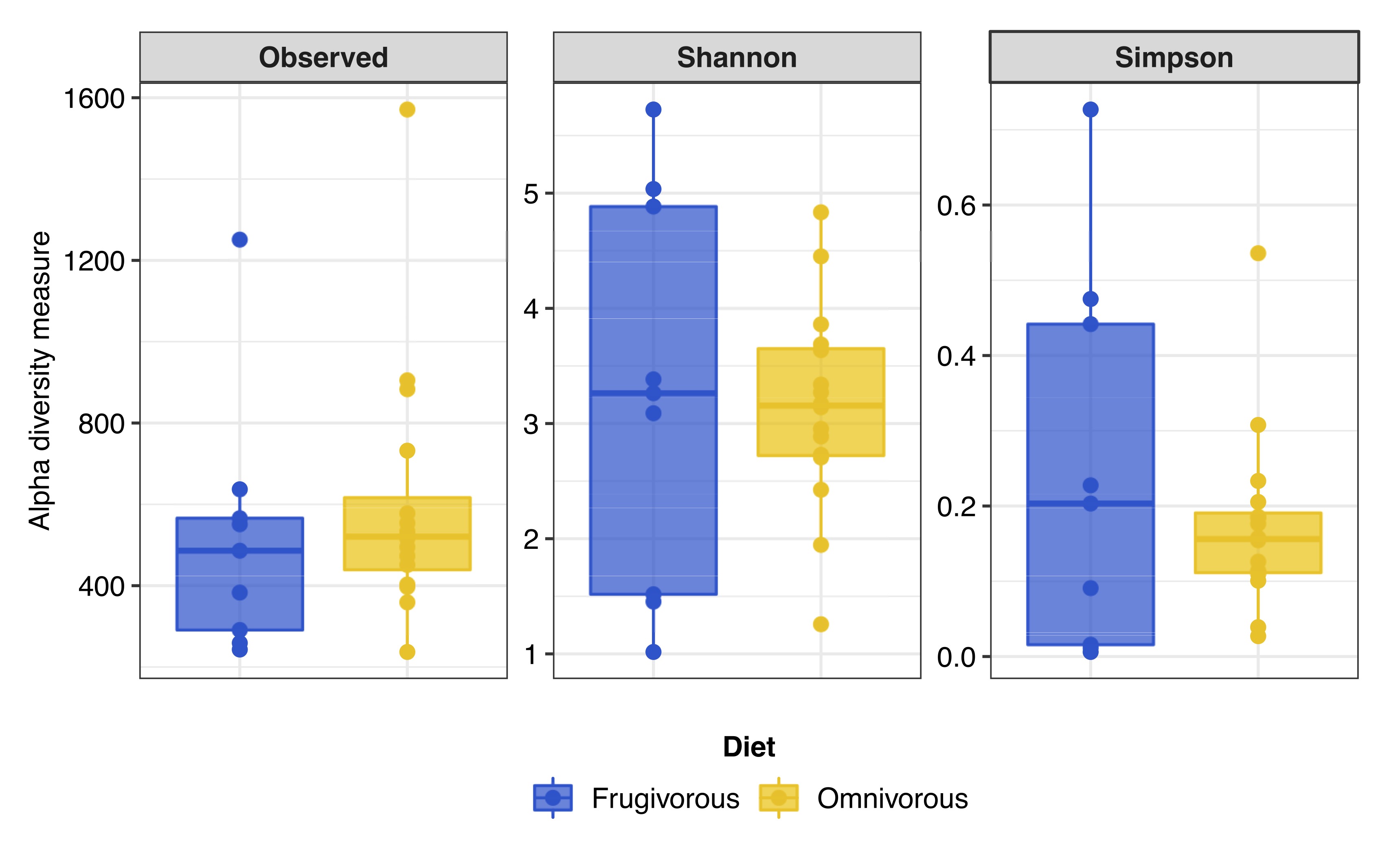

Supplement: Supplemental Information 3 [file peerj-11-15169-s003.jpg]

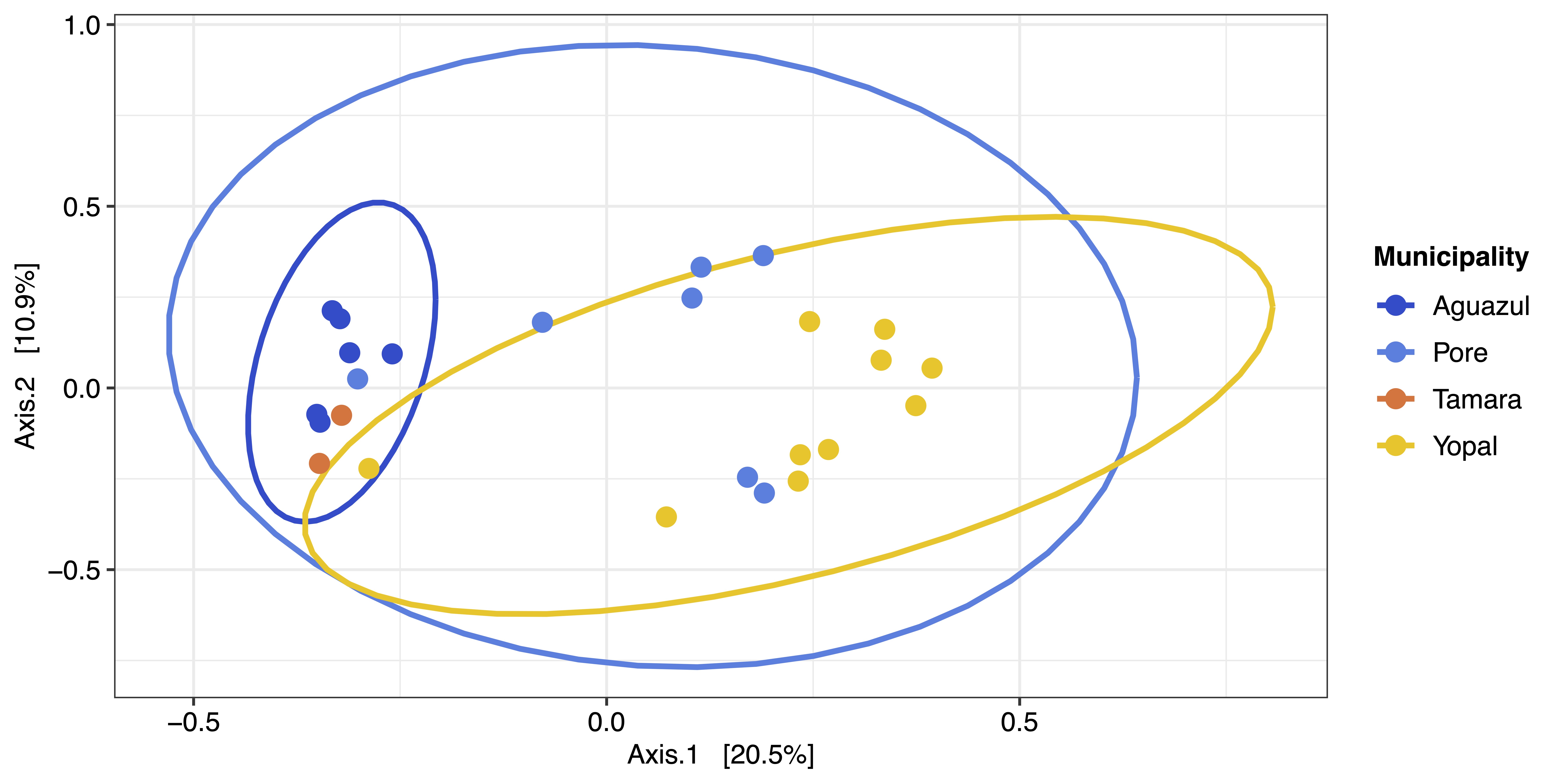

Supplement: Supplemental Information 4 [file peerj-11-15169-s004.jpg]

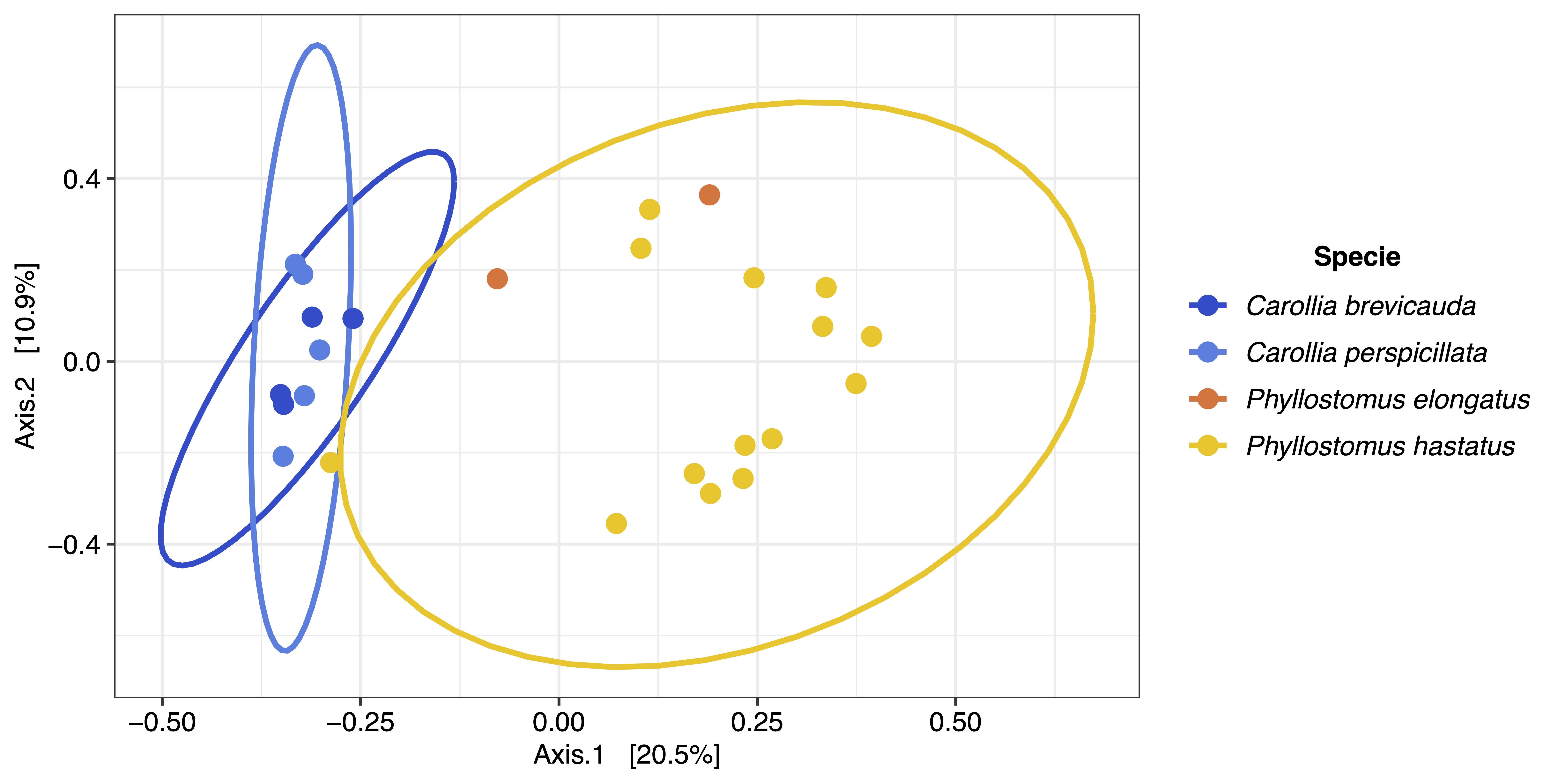

Supplement: Supplemental Information 5 [file peerj-11-15169-s005.jpg]
